# IL15RA-STAT3-GPX4/ACSL3 signaling leads to ferroptosis resistance in pancreatic cancer: IL15RA-STAT3-GPX4/ACSL3 leads to ferroptosis resistance in PC

**DOI:** 10.3724/abbs.2024153

**Published:** 2024-10-12

**Authors:** Di Wu, Zhiliang Wang, Yue Zhang, Yang Yang, Yue Yang, Guangchen Zu, Xianjun Yu, Weibo Chen, Yi Qin, Xiaowu Xu, Xuemin Chen

**Affiliations:** 1 Department of Hepatopancreatobiliary the Third Affiliated Hospital of Soochow University Changzhou 213000 China; 2 Department of Pancreatic Surgery Fudan University Shanghai Cancer Center Shanghai 200032 China; 3 Shanghai Pancreatic Cancer Institute Shanghai 200032 China; 4 Pancreatic Cancer Institute Fudan University Shanghai 200032 China

**Keywords:** pancreatic stellate cell, pancreatic cancer, ferroptosis, IL15, IL15RA

## Abstract

Pancreatic ductal adenocarcinoma (PDAC) is a highly malignant disease with a poor prognosis, and the lack of effective treatment methods accounts for its high mortality. Pancreatic stellate cells (PSCs) in the tumor microenvironment play an important role in the development of PDAC. Previous studies have reported that patients with PDAC are more vulnerable to ferroptosis inducers. To investigate the relationship between PSCs and pancreatic cancer cells, a coculture system is used to further reveal the influence of PSCs on ferroptosis resistance in PDAC using many *in vitro* and *in vivo* experiments. Our results show that PSCs promote ferroptosis resistance in pancreatic cancer cells. We further demonstrate that IL15 secretion by PSCs activates the IL15RA-STAT3-GPX4/ACSL3 axis. The simultaneous upregulation of GPX4 and ACSL3 prevents lipid peroxidation and ultimately protects pancreatic cancer cells from ferroptosis both *in vitro* and *in vivo*. This study demonstrates that PSCs protect pancreatic cancer cells in a paracrine manner and may indicate a novel strategy for the treatment of PDAC.

## Introduction

Pancreatic ductal adenocarcinoma (PDAC) is a highly malignant disease with a poor prognosis, and the lack of effective treatment methods accounts for its high mortality. Pancreatic stellate cells (PSCs) in the tumor microenvironment play an important role in the development of PDAC. Patients with PDAC urgently need effective treatment because the prognosis is very poor, and the 5-year survival rate is only 10% [1].

Ferroptosis is a unique form of cell death resulted from the overwhelming iron-dependent accumulation of lethal amounts of ROS [2]. Although ferroptosis is a promising strategy in various drug-resistant tumors, cancer cells have developed multiple resistance mechanisms to protect themselves from ferroptosis. Ferroptosis has been widely studied in ovarian cancer, breast cancer, liver cancer, rectal cancer and lung cancer. However, few studies have investigated ferroptosis in pancreatic cancer. Recently, the role of ferroptosis in pancreatic cancer has attracted the attention of researchers. Inducing ferroptosis in pancreatic cancer cells may inhibit tumor growth and reduce tumor chemoresistance.

PDAC has a dismal prognosis, perhaps partially because severe desmoplastic reaction may prevent classic chemotherapeutic drugs from accessing tumor cells [3]. PSCs are the main cells that cause desmoplastic reactions in patients with PDAC [ 4, 5]. However, the relationship between parenchymal cells and stromal cells is far greater [ 6, 7]. To date, several types of cell death, including apoptosis, autophagy, necroptosis, pyroptosis, and ferroptosis, have been described [2]. Recently, several reports revealed that PDAC is more vulnerable to ferroptosis inducers [8]. However, the underlying mechanisms of ferroptosis resistance in pancreatic cancer mediated by stromal cells in the tumor microenvironment remain unknown.

In the present study, we aimed to investigate the relationship between PSCs and pancreatic cancer cells and to determine whether an underlying mechanism can partially explain ferroptosis resistance in PDAC. Our results revealed that activated PSCs secrete IL15, which upregulates the expression of IL15RA in pancreatic cancer cells via a paracrine signaling pathway.

## Materials and Methods

### Cell culture and treatment

The human pancreatic cancer cell lines PANC-1 and SW1990 were obtained from the American Type Culture Collection (ATCC; Manassas, USA) and were passaged in our laboratory fewer than 6 months after receipt. PANC-1 cells were cultured in Dulbecco’s modified Eagle’s medium (DMEM; HyClone, Carlsbad, USA) supplemented with 10% FBS (Gibco, Carlsbad, USA). SW1990 cells were cultured in L15 medium (HyClone) supplemented with 10% FBS. Penicillin (100 U/mL) and streptomycin (0.1 mg/mL) were added, and the culture medium was changed every 2 days. PSCs were isolated and cultured according to previous methods [1].

### Reagents and antibodies

All-trans retinoic acid (ATRA; No. S1653), erastin (No. S7242) and Stattic (No. S7024) were purchased from Selleck Chemicals (Houston, USA). C11-Bodipy (No. D3861) was purchased from Thermo Fisher Scientific (Waltham, USA). IL-15 (No. 200-15) was purchased from PeproTech (Shanghai, China). Signal transducer and activator of transcription 3 (STAT3; No. sc-8019) and phosphorylated (p-STAT3) (pY705; No. sc-8059) antibodies were purchased from Santa Cruz (Santa Cruz, USA). Glutathione peroxidase 4 (GPX4; No. A11243) and IL-15 (No. A19319) antibodies were purchased from ABclonal (Shanghai, China). Acyl-CoA synthetase long chain family member 3 (ACSL3; No. D361226) and IL-15 receptor subunit alpha (IL15RA; No. D160511) antibodies were purchased from BBI Life Sciences (Shanghai, China). β-Actin (No. AF7018), goat anti-rabbit IgG (H+L) HRP (No. S0001) and goat anti-mouse IgG (H+L) HRP (No. S0002) were purchased from Affinity (Cincinnati, USA). Alpha smooth muscle actin (α-SMA; No. ab124964), goat anti-rabbit IgG H&L (Alexa Fluor® 647; No. ab150079) and vimentin (No. ab16700) antibodies were purchased from Abcam (Cambridge, UK). Fibroblast activation protein (FAP; No. #66562) and STAT3 (No. 9139S) antibodies were purchased from Cell Signaling Technology (Danvers, USA).

### siRNA treatments

The procedure for siRNA transfection was performed according to the instructions of the kit using Lipofectamine 3000 (Invitrogen, Carlsbad, USA). In brief, siRNA duplexes against IL15 were transfected into PSCs. The siRNA duplexes were purchased from RIBOBIO Co., Ltd. (Guangzhou, China), and the sequences used were as follows: si-IL15-1: 5′-GCATATTGATGCTACTTTA-3′; si-IL15-2: 5′-GGGTGAATGTAATAAGTGA-3′; and si-NC: 5′-UUCUCCGAACGUGUCACGUTT-3′. siRNA duplexes against IL15RA were transfected into pancreatic cancer cells. The siRNA sequences used were as follows: si-IL15RA-1: 5′-GGCCAGCGTTGAAATGGAA-3′; si-IL15RA-2: 5′-GCGGTACATTTGTAACTCT-3′; and si-NC: 5′-UUCUCCGAACGUGUCACGUTT-3′.

### Construction of *IL15RA*-knockout SW1990 cell line

Guide RNA (gRNA) sequences targeting the exon regions of the human *IL15RA* gene were designed by the Broad Institute GPP tool. The sequences used were as follows: IL15RA-1: 5′-ACAACAGCAGCTATTGTCCC-3′; and IL15RA-2: 5′-TCCCAGCTCAAACAACACAG-3′. The designed gRNA sequences were cloned and inserted into a lentiviral CRISPR/Cas9 vector, lentiCRISPR v2, allowing for the coexpression of Cas9 nuclease and gRNA. Recombinant lentiviruses can then be produced by cotransfecting the lentiCRISPR vector along with packaging plasmids into HEK293T cells. After the viral supernatant was harvested and concentrated, SW1990 cells were transduced with lentiviral particles containing IL15RA-specific gRNA and Cas9. The stably infected cells were subsequently selected with puromycin for further use. Finally, the knockout efficiency was validated by western blot analysis.

### Western blot analysis

Western blot analysis was carried out as previously described [9]. Briefly, whole-cell protein lysates were extracted with RIPA lysis buffer containing protease and phosphatase inhibitors (No. P1050; Beyotime, Shanghai, China), separated by SDS-AGE and blotted onto polyvinylidene fluoride membranes (Bio-Rad, Hercules, USA). After being blocked, the membranes were incubated with the corresponding antibodies (IL15RA: 1:1000, p-STAT3: 1:500, STAT3: 1:500, GPX4: 1:1000, ACSL3: 1:1000 and β-actin: 1:1000) at 4°C overnight. Next, the membranes were incubated with HRP-conjugated secondary antibodies [goat anti-rabbit IgG (H+L) HRP: 1:2000, or goat anti-mouse IgG (H+L) HRP: 1:2000]. Finally, the signals of the immunoblots were developed with an enhanced chemiluminescence (ECL) system (Millipore, Billerica, USA) and images were captured with a Tanon 5200 Chemiluminescent Imaging System (Shanghai, China).

### Immunofluorescence staining

After fixation with polyformaldehyde for 30 min, the samples were blocked with 5% BSA and treated with 0.1% Triton X-100 to increase cell membrane permeability. The samples were subsequently incubated with primary antibodies (α-SMA: 1:500) for 12 h at 4°C. Then, secondary antibodies (goat anti-rabbit IgG H&L Alexa Fluor® 647: 1:500) were used. Images were captured with a fluorescence microscope (Leica, Solms, Germany).

### RNA isolation and quantitative real-time PCR

Quantitative real-time PCR was carried out as previously described [ 10, 11]. Briefly, total RNA was extracted using an RNA Purification kit (No. B004D; EZBioscience, Shanghai, China). cDNA was obtained by reverse transcription using the TaKaRa PrimeScript RT reagent kit (No. RR036A; TaKaRa, Dalian, China). Quantitative real-time PCR was conducted with a QuantStudio 6 Flex Real-Time PCR system (Thermo Fisher Scientific). The primers used are shown in Table 1.

**Table:** **Table 1** Sequences of primers used for real-time PCR in this study

Gene	Primer sequence (5′→3′)
*α-SMA*	Forward	CTATGCCTCTGGACGCACAACT
	Reverse	CAGATCCAGACGCATGATGGCA
*FAP*	Forward	GGAAGTGCCTGTTCCAGCAATG
	Reverse	TGTCTGCCAGTCTTCCCTGAAG
*Collagen I*	Forward	GATTCCCTGGACCTAAAGGTGC
	Reverse	AGCCTCTCCATCTTTGCCAGCA
*Fibronectin*	Forward	ACAACACCGAGGTGACTGAGAC
	Reverse	GGACACAACGATGCTTCCTGAG’
*Laminin B1*	Forward	GAGGTGTCTCAAGTGCCTGTAC
	Reverse	ACTGGCAGTCAGAGCCGTTACA
*IL15RA*	Forward	TGGCTATCTCCACGTCCACTGT
	Reverse	CATGGCTTCCATTTCAACGCTGG
*GPX4*	Forward	AAGTTCAGTCAGAGACCTGCG
	Reverse	ATATCCGAGCCCTCCTCCTTC
*ACSL3*	Forward	GCCGAGTGGATGATAGCTGC
	Reverse	ATGGCTGGACCTCCTAGAGTG
*β-Actin*	Forward	CACCATTGGCAATGAGCGGTTC
	Reverse	AGGTCTTTGCGGATGTCCACGT’

### EdU assay

The EdU assay was performed using an EdU Cell Proliferation kit with Alexa Fluor 594 (No. C0078L; Beyotime) according to the manufacturer’s instructions. Briefly, pancreatic cancer cells were seeded in 96-well plates at 1 × 10 ^4^ cells/well. After being cultured with medium containing 10 μM EdU for 2 h, the cells were washed with PBS and fixed with polyformaldehyde. Triton X-100 (0.5%) was used to increase cell membrane permeability. The cells were incubated with Click reaction mixture, followed by Hoechst staining. Each well was randomly imaged in five fields under a fluorescence microscope (Leica).

### Cell viability assay

Cell viability was assessed using Cell Counting Kit-8 (CCK-8; No. C0037; Beyotime). Briefly, cells (4000 cells/100 μL) were plated in 96-well plates, and the absorbance was measured at 450 nm at different time points (0, 24, 48, 72, and 96 h) with a microplate reader (Thermo Fisher Scientific).

### Colony formation assay

Pancreatic cancer cells (500 cells/well) were seeded in each well. The culture medium was changed every 2 days for two weeks. Then, the cells were washed twice with PBS, and fixed with polyformaldehyde, and stained with a 0.3% crystal violet solution for 30 min at room temperature. The number of colonies was photographed and counted.

### Oil Red staining

Cells were fixed with 4% paraformaldehyde for 1 h and were then stained with Oil Red O (Beyotime) for 1 h according to the manufacturer’s instruction, followed by a final wash with washing buffer. Cells were observed and photographed using a microscope (Nikon, Tokyo, Japan).

### ELISA

Conditioned media were collected from 3 separate experiments. The IL-15 levels in the culture supernatants were measured using the Human IL-15 ELISA kit (No. BY-EH110341; BYabscience, Nanjing, China) according to the manufacturer’s instructions.

### C11-BODIPY staining

Cells were plated in 6-well cell culture plates and then incubated with C11-bodipy contained culture medium at a concentration of 2 μM for 30 min. Then, cells were washed with PBS twice and resuspended. Fluorescence intensity was detected by flow cytometry (Beckman, Pasadena, USA) and results were analyzed by FlowJo software. For confocal imaging, cells were plated in round coverslip. Before detection, cells were incubated with 2 μM C11-bodipy for 30 min. Next, cells were washed with PBS twice and images were acquired using a confocal microscope (Nikon).

### Malondialdehyde (MDA) assay

Cells were plated in 6-well cell culture plates. Protein concentrations were measured using Enhanced BCA Protein Assay kit (No. P0009; Beyotime) and MDA was detected using Lipid Peroxidation MDA Assay kit (No. S0131S; Beyotime) according to the manufacturer’s instructions.

### Reactive oxygen species (ROS) assay

Intracellular ROS levels were detected with Reactive Oxygen Species Assay kit (No. S0033S; Beyotime). Briefly, cells were stained with 2.5 μM DCFH-DA for 30 min, and washed twice with PBS. After washing, fluorescence intensity was detected by flow cytometry (Beckman) and results were analyzed by FlowJo software.

### GSH/GSSG assay and glutathione peroxidase (GPX) activity assay

The ratio of GSH/GSSG was measured using GSH and GSSG Assay kit (No. S0053; Beyotime) according to the manufacturer’s instruction. Protein concentrations of the cells lysates were measured and used for normalization. GPX activity was measured using GPX Activity Assay kit (No. D799618; Sangon, Shanghai, China) according to the manufacturer’s instruction.

### Transmission electron microscope (TEM) imaging

Cells were resuspended and washed with PBS twice. Then, cells were fixed with 2.5% glutaraldehyde. TEM imaging was conducted by Servicebio (Wuhan, China). Briefly, samples were dehydrated at room temperature and underwent resin penetration and embedding. After positioning, ultrathin section and staining, samples were finally observed under a TEM.

### RNA-seq and lipidomic analysis

RNA-seq and LS-MS/MS-based untargeted lipidomic profiling were conducted at BioProfile Biotechnology (Shanghai, China). Briefly, total RNA was isolated using Trizol Reagent (Invitrogen), after which its concentration, quality and integrity were determined via a NanoDrop spectrophotometer (Thermo Fisher Scientific). Three micrograms of RNA were used as input material for the RNA sample preparations. The sequencing libraries were generated according to the following steps. First, mRNA was purified from total RNA using poly-T oligo-attached magnetic beads. Fragmentation was carried out using divalent cations under elevated temperature in Illumina proprietary fragmentation buffer. First-strand cDNA was synthesized using random oligonucleotides and Super Script II. Second-strand cDNA synthesis was subsequently performed using DNA polymerase I and RNase H. The remaining overhangs were converted into blunt ends via exonuclease/polymerase activities, and the enzymes were removed. After adenylation of the 3′ ends of the DNA fragments and Illumina PE adapter oligonucleotides were ligated to prepare for hybridization. To select cDNA fragments of the preferred 400‒500 bp in length, the library fragments were purified via the AMPure XP system (Beckman Coulter, Beverly, USA). DNA fragments with ligated adaptor molecules on both ends were selectively enriched using Illumina PCR Primer Cocktail in a 15-cycle PCR. Products were purified (AMPure XP system) and quantified using the Agilent high-sensitivity DNA assay on a Bioanalyzer 2100 system (Agilent, Santa Clara, USA). The sequencing library was then sequenced on a NovaSeq 6000 platform (Illumina) at Shanghai Bioprofile Technology Co., Ltd. (Shanghai, China). Lipidomic profiling was conducted using the ultra-performance liquid chromatography-mass spectrometry system (Q Exactive Plus; Thermo Fisher Scientific) at Shanghai BioProfile Biotechnology (Shanghai, China). The mass spectrometric data were analyzed using the LipidSearch 4.1.30 software (Thermo Fisher Scientific).

### Mitochondrial membrane potential (MMP) measurement

MMP was measured using an enhanced mitochondrial membrane potential assay kit with JC-1 (No. C2003S; Beyotime). Briefly, after 30 min incubation with 1:200 JC-1 working buffer at 37°C, the fluorescence images of cells were observed and photographed using a confocal microscope.

### Apoptosis analysis

The apoptotic status of the cells was detected using an FITC Annexin V Apoptosis Detection Kit I (BD, Cat. No. 556547). Briefly, cells were incubated with FITC Annexin V in buffer containing propidium iodide (PI) and analyzed by flow cytometry. The results were analyzed by FlowJo software.

### Chromatin immunoprecipitation (ChIP) and dual luciferase reporter assay

ChIP was performed using the Chromatin Immunoprecipitation (ChIP) kit (No. Bes5001; BersinBio, Shangai, China). Briefly, pancreatic cancer cells were seeded in 10-cm dishes, crosslinked with the reagent when they grew to 90% confluence, and lysed with SDS buffer. Then, ultrasound was used to break the DNA into fragments of 200–600 bp, and specific antibodies or anti-human IgG antibody was used to pull down the DNA. After wash with high salt and low salt buffers, DNA was eluted and decrosslinked, and enriched sequences were examined by qPCR. *GPX4* and *ACSL3* primers used in chip assay are shown in Table 2.

**Table:** **Table 2** Sequences of primers used for ChIP in this study

Gene	Primer sequence (5′→3′)
*GPX4-1*	Forward	GCAGTGAGCTGAGATAGCGC
	Reverse	TGTAGACATGGGGGTCTCGC
*GPX4-2*	Forward	CTGAGGCTGGAGGATCACTTG
	Reverse	CAGAAAAGTGTCCCCAACTC
*ACSL3-1*	Forward	TCATATTGGTCAGGCTGGTC
	Reverse	TTGCTGTTTAACAAGCTAAG
*ACSL3-2*	Forward	ACGAAAAAGAATATGCATAC
	Reverse	CCGGACCTGGCTGCAGTCTG

The GPX4 and ACSL3 promoter region, spanning from ‒2000 to +200 of the transcription start site, was cloned and inserted into the pGL3-Basic vector (Promega, Madison, USA). The coding sequence of human *STAT3* was cloned and inserted into the pCMV-c-flag vector (Beyotime) to generate STAT3 expression plasmids. A dual-luciferase system (No. E1910; Promega) was used to measure Firefly and Renilla luciferase activities according to the manufacturer’s protocol.

### *In vivo* tumor model

Animal experiment protocols were approved by the Animal Care and Use Committee of the Third Affiliated Hospital of Soochow University (Changzhou, China). The SW1990 cell line and PSCs were used to establish the *in vivo* tumor model. Briefly, SW1990 cells were resuspended in a serum-free medium with or without the mixture of PSCs at a density of 1 × 10 ^8^ cells/mL. Next, female BALB/c nude mice (4 weeks old; purchased from Shanghai SLAC Laboratory Animal Co. Ltd, Shanghai, China) were randomly divided into 4 groups. For group 1, *in situ* injection of 40 μL cell suspension containing 4 × 10 ^6^ SW1990 cells were performed. For group 2, group 3 and group 4, *in situ* injection of 50 μL cell suspension containing 4 × 10 ^6^ SW1990 cells and 1 × 10 ^6^ PSCs were performed. After 4 weeks, tumors were harvested and tumor volume was calculated using the formula as follows: tumor volume = length × width ^2^/2.

### Immunohistochemical (IHC) staining

IHC staining was performed as described previously [12]. Briefly, the paraffin slides were dewaxed, rehydrated, and subjected to antigen retrieval in sodium citrate buffer (pH 6.0) or Tris-EDTA buffer (pH 9.0). IHC was performed using a streptavidin-peroxidase kit (ZSGB-BIO, Beijing, China) according to the manufacturer’s instructions. After visualization with a DAB kit (Solarbio, Beijing, China), the slides were counterstained with hematoxylin [12]. The following antibodies were used: anti-IL15RA (1:100), anti-GPX4 (1:100), and anti-ACSL3 (1:50).

### Statistical analysis

Statistical analyses of the data were performed using GraphPad Prism 8.0 and SPSS 24.0. All results were presented as the mean ± standard error mean (SEM). The unpaired two-tailed Student’s *t* test was used for two group comparisons. *P*  < 0.05 was considered statistically significant.

## Results

### PSCs can be activated by pancreatic cancer cells upon coculture

To assess whether PSCs can be activated by pancreatic cancer cells, we used the coculture system as described previously [1]. After 3 days of coculture, PSCs were harvested. Cellular immunofluorescence demonstrated the upregulation of α-SMA ( Supplementary Figure S1A). Oil Red staining showed reduced lipid droplets upon coculture, which demonstrated the activation state of PSCs ( Supplementary Figure S1B). The protein levels of FAP, Vimentin and α-SMA were upregulated as determined by western blot analysis ( Supplementary Figure S1C). Furthermore, qRT-PCR revealed the upregulated levels of *α-SMA*, *FAP*, *Collagen I*, *Fibronectin* and *Laminin B1* ( Supplementary Figure S1D).

### Activated PSCs promote the proliferation ability and gemcitabine resistance of pancreatic cancer cells

**Figure 1:**
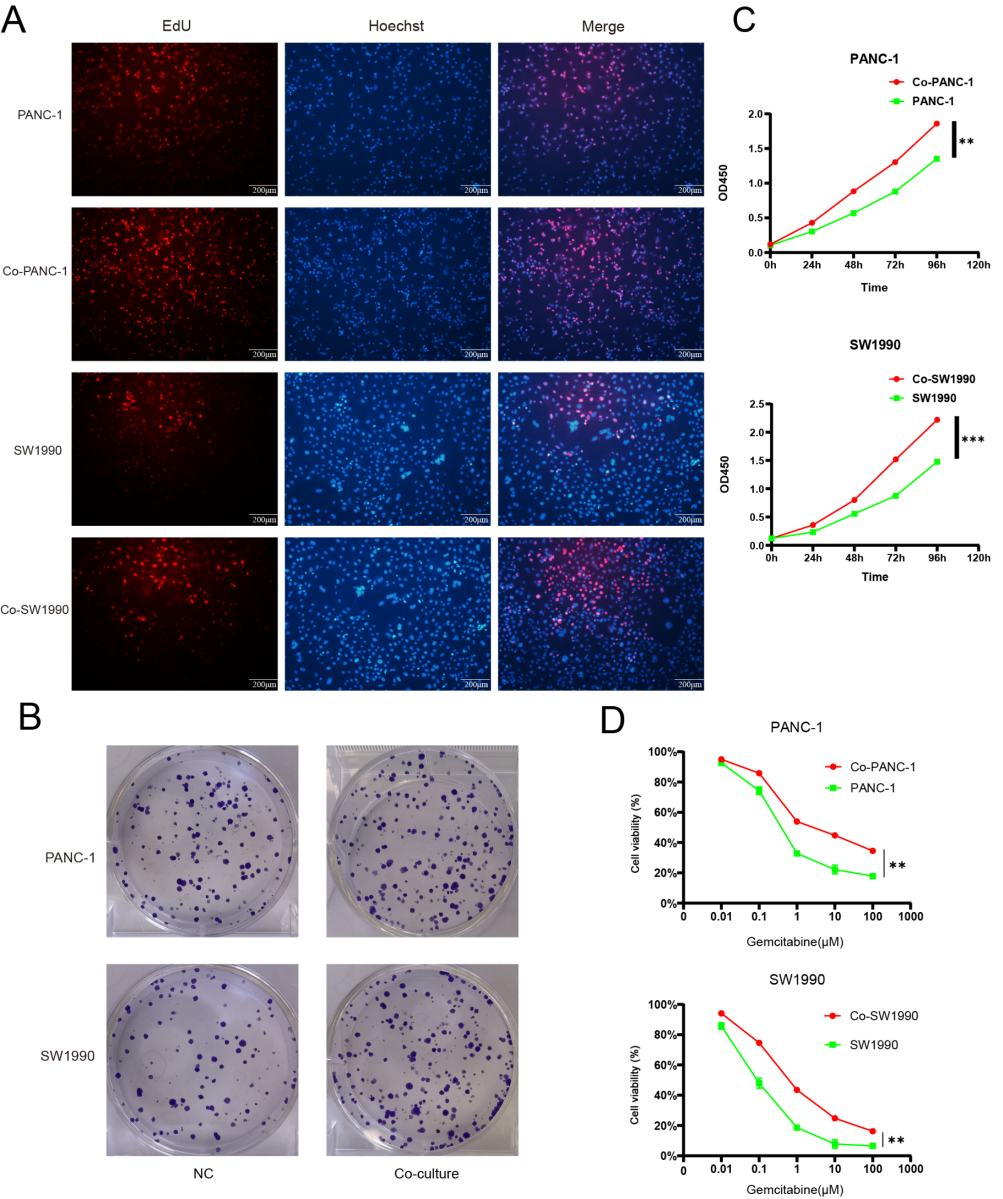
Figure 1 Activated PSCs promote the proliferation ability and gemcitabine resistance of pancreatic cancer cells (A) EdU assay demonstrated that the proportion of proliferative pancreatic cancer cells increases after coculture. Scale bar: 200 μm. (B) Colony formation assay revealed that more colonies formed after coculture. (C) CCK-8 revealed that cell viability of pancreatic cancer cells increased after cocultured with PSCs. (D) Remarkable increase in gemcitabine resistance. **P < 0.01, ***P < 0.001.

To assess the influence of activated PSCs on pancreatic cancer cells, their proliferation ability was assessed by EdU assay ( Figure 1A), colony formation assay ( Figure 1B) and cell viability assay ( Figure 1C). The results revealed that the proliferative ability of pancreatic cancer cells was enhanced by activated PSCs. To test whether activated PSCs can promote gemcitabine resistance, a cytotoxicity experiment was carried out, and the results revealed a remarkable increase in gemcitabine resistance ( Figure 1D).

### Activated PSCs increase the GSH/GSSG ratio and GPx activity of pancreatic cancer cells

To assess whether activated PSCs influence the GSH/GSSG ratio, which serves as a ferroptosis inhibitor, the GSH/GSSG ratio was tested. Moreover, the GPx activity of pancreatic cancer cells was tested with or without activated PSCs. The results showed that upon coculture, the GSH/GSSG ratio ( Supplementary Figure S2A) and GPx activity ( Supplementary Figure S2B) of pancreatic cancer cells were increased.

### IL15RA, ACSL3 and GPX4 expressions in pancreatic cancer cells are upregulated upon cocultured with activated PSCs

To determine whether activated PSCs can modify the gene expression of pancreatic cancer cells, RNA-Seq was performed. Upon coculture, the number of upregulated genes was much more than the number of downregulated genes in pancreatic cancer cells ( Supplementary Figure S3A). Boxplots demonstrated the higher expressions of IL15RA, ACSL3 and GPX4 in a more direct way ( Supplementary Figure S3B). The upregulated levels of IL15RA, ACSL3 and GPX4 in pancreatic cancer cells were verified by qRT-PCR ( Supplementary Figure S3C) and western blot analysis ( Supplementary Figure S3D).

### Activated PSCs increase the content of PC-MUFA and PE-MUFA

MUFA-containing phosphatidylcholine (PC) and MUFA-containing phosphatidylethanolamine (PE) result in the resistance to lipid peroxidation and ferroptosis. Meanwhile, ACSL3 has been reported to be involved in regulating the conversion of free long-chain fatty acids (MUFAs) to acyl CoA esters [13]. We then measured the amount of PC-MUFA and PE-MUFA, and the results showed that activated PSCs increased the contents of PC-MUFA and PE-MUFA ( Supplementary Figure S4A,B), which is consistent with the result of ACSL3 expression.

### ATRA reverses the activation state of PSCs and reduces the secretion of IL15

To determine whether ATRA affects the activation of PSCs, cells were treated with culture medium containing ATRA (10 μM) for 10 days. Oil Red O staining was subsequently performed, and the results revealed more lipid droplets after ATRA treatment ( Supplementary Figure S5A). qRT-PCR revealed that after treatment with ATRA, the *α-SMA*, *FAP*, *Collagen I*, *Fibronectin* and *Laminin* *B1* levels were decreased ( Supplementary Figure S5B). Western blot analysis revealed that ATRA reduced FAP, α-SMA, Vimentin and IL15 expression levels ( Supplementary Figure S5C). To determine whether the activation state of PSCs influences the secretion of IL15, its levels in the culture supernatants were measured using the Human IL15 ELISA kit. The results revealed that the secretion of IL15 was also decreased ( Supplementary Figure S5D).

### Activated PSCs promote ferroptosis resistance in pancreatic cancer cells

**Figure 2:**
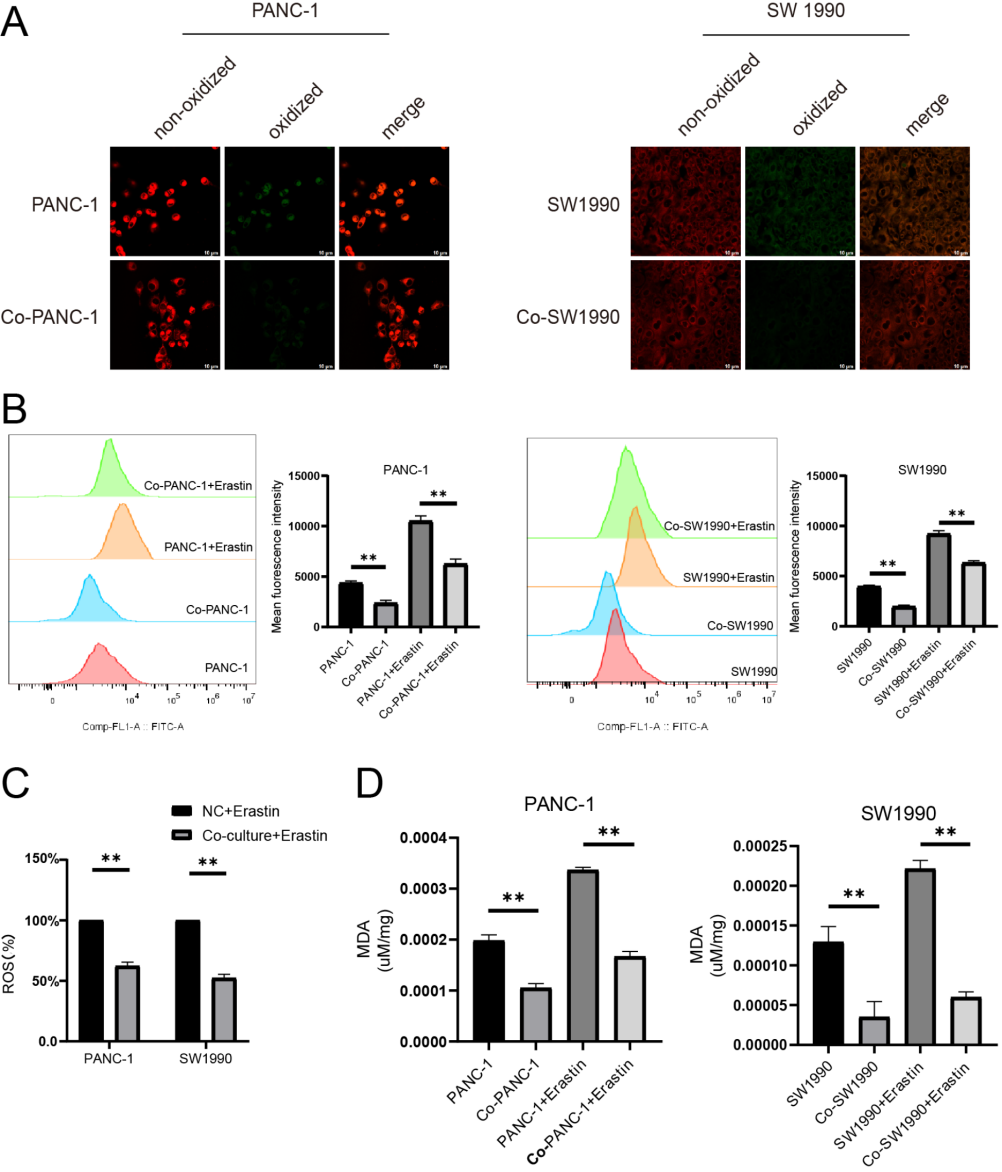
Figure 2 Activated PSCs promote pancreatic cancer cell ferroptosis resistance (A) Confocal imaging revealed the effect of activated PSCs on lipid peroxidation of pancreatic cancer cells. Scale bar: 10 μm. (B) BODIPY 581/591C11 was used to detect lipid peroxidation level. (C) ROS levels were detected in pancreatic cancer cells in the presence or absence of activated PSCs. (D) Concentrations of MDA were detected in pancreatic cancer cells in the presence or absence of activated PSCs. **P < 0.01.

Ferroptosis sensitivity of pancreatic cancer cells with or without activated PSCs was assessed. BODIPY 581/591 C11 probe was used to detect oxidized lipid and further analyzed by flow cytometry and confocal microscopy. Fluorescence would change from red to green when lipid peroxidation occurred and confocal microscopy revealed that activated PSCs caused a decrease of lipid peroxidation level in pancreatic cancer cells upon coculture ( Figure 2A). Flow cytometry analysis also revealed a decrease in lipid peroxidation level ( Figure 2B). Intracellular ROS levels were detected and the result demonstrated that activated PSCs could decrease the intracellular ROS level ( Figure 2C). This result was further confirmed by the phenomenon that activated PSCs also decreased the level of MDA (malondialdehyde), the end product of lipid peroxidation ( Figure 2D).

### Activated PSCs protect the function of mitochondria in pancreatic cancer cells when subjected to erastin exposure

**Figure 3:**
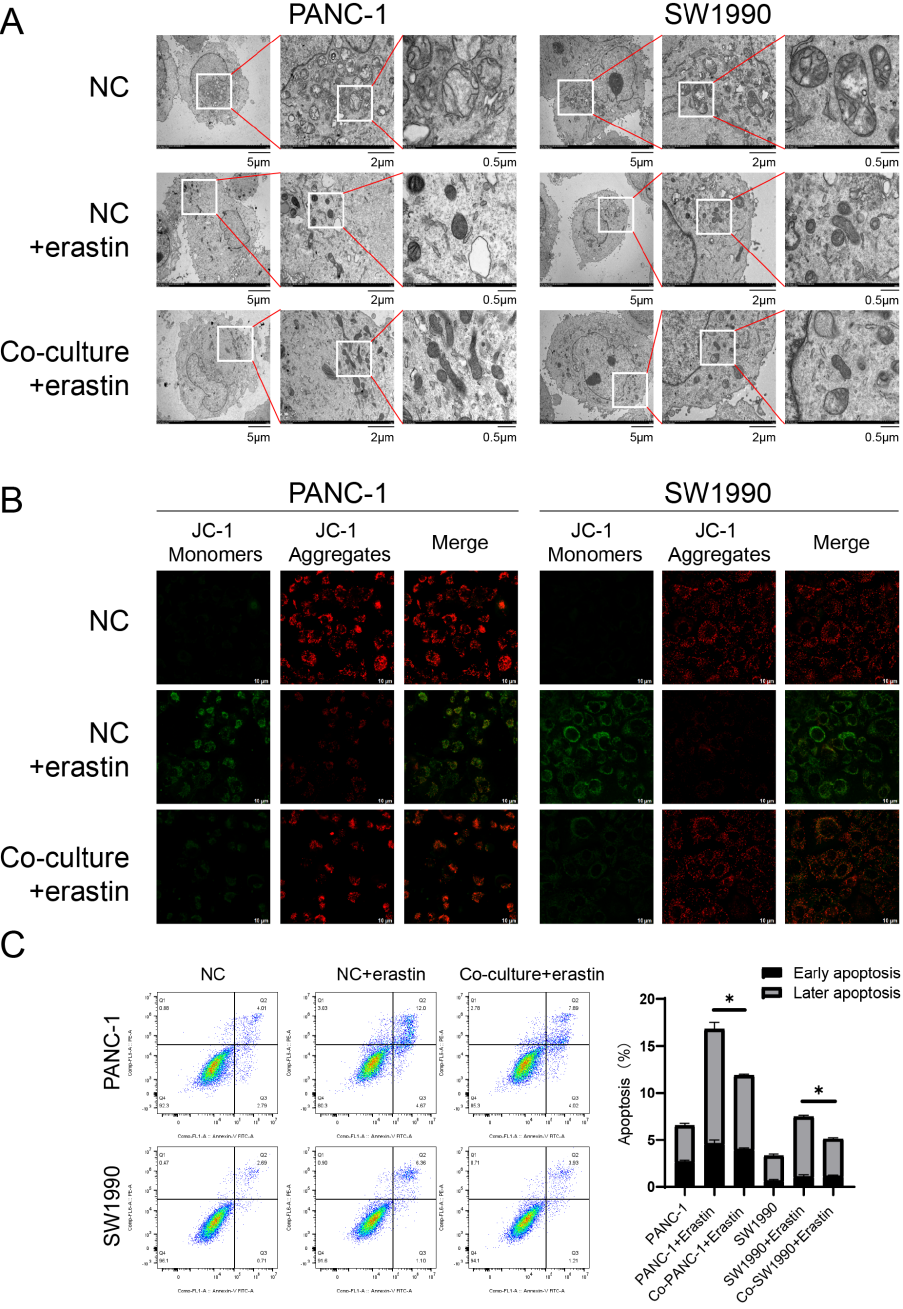
Figure 3 Activated PSCs protect the function of mitochondria in pancreatic cancer cells and reduce mitochondria-dependent apoptosis in pancreatic cancer cells (A) TEM imaging was conducted in pancreatic cancer cells to observe alterations of mitochondrial morphology. (B) JC-1 assay was performed to detect MMP changes. Scale bar: 10 μm. (C) Apoptosis was analyzed in pancreatic cancer cells. *P < 0.05.

Normal structure is vital for maintaining mitochondrial functions. To further investigate the function of mitochondria with or without activated PSCs, we treated pancreatic cancer cells with erastin, a ferroptosis activator, to determine whether activated PSCs could alleviate mitochondrial damage. TEM revealed that after erastin treatment, there was obvious mitochondrial damage and mitochondrial shrinkage, which could be reversed by coculture with activated PSCs ( Figure 3A). The MMP was measured via the JC-1 assay. The cells in green indicate a low MMP, whereas those in red indicate a high MMP. As shown in Figure 3B, after treatment with erastin, the MMP in pancreatic cancer cells decreased, and this effect could also be rescued by coculture with activated PSCs.

### Activated PSCs reduce mitochondria-dependent apoptosis in pancreatic cancer cells

Erastin enhanced mitochondria-dependent apoptosis. However, this effect was alleviated by the coculture of activated PSCs ( Figure 3C), suggesting that activated PSCs reduced the apoptotic rate by recover part of the mitochondrial dysfunction induced by intracellular ROS.

### Activated PSCs mediate ferroptosis resistance in pancreatic cancer cells and promote cell proliferation by the secretion of IL15

**Figure 4:**
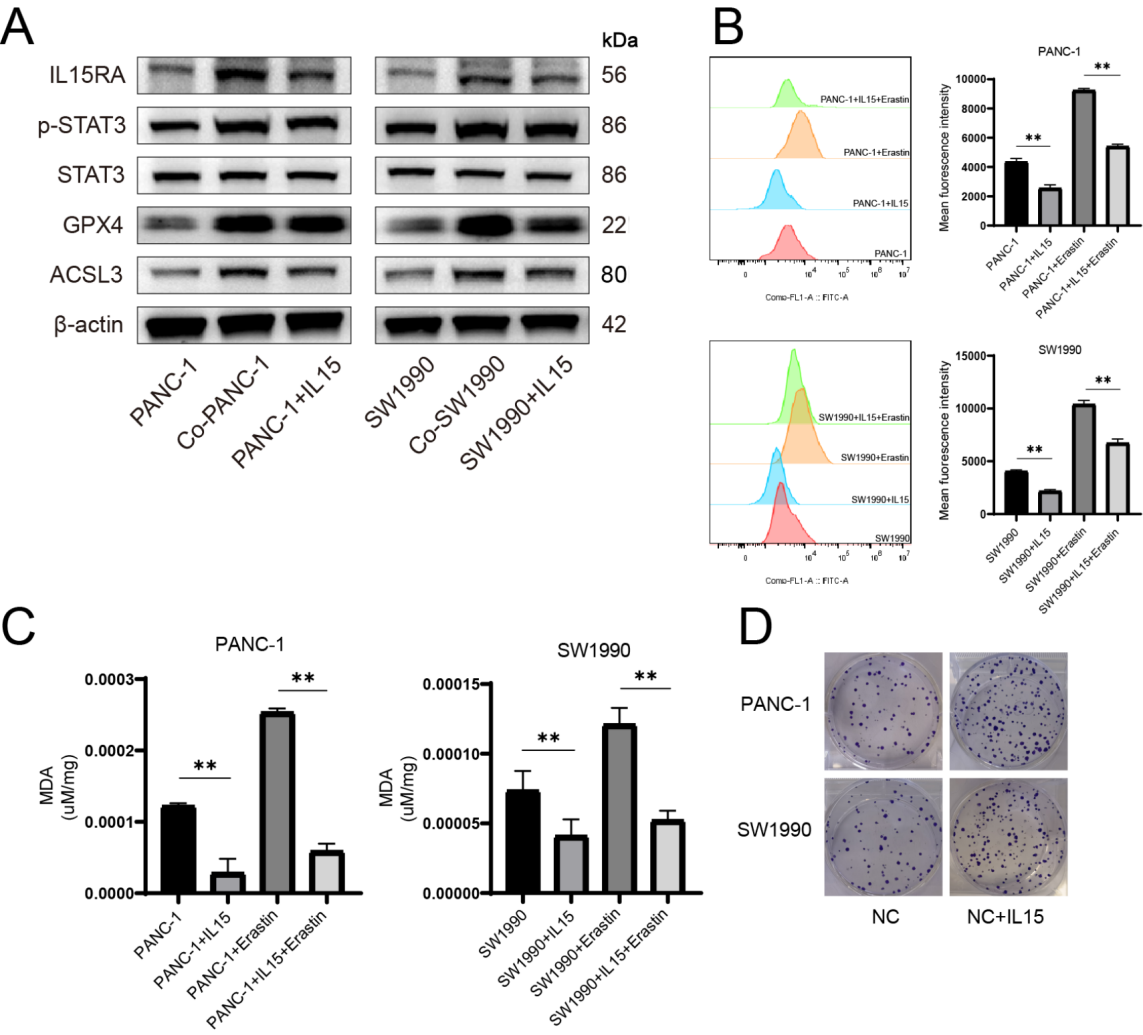
Figure 4 IL15 promotes cell proliferation and mediates ferroptosis resistance in pancreatic cancer cells (A) Pancreatic cancer cells were pretreated with IL15, and the protein levels of IL15RA, p-STAT3, STAT3, GPX4 and ACSL3 were detected. (B) BODIPY 581/591C11 was used to detect lipid peroxidation level. (C) Concentration of MDA was detected in pancreatic cancer cells with or without IL15. (D) Colony formation assay was performed to detect the proliferation ability of pancreatic cancer cells. **P < 0.01.

To determine whether IL15 can promote ferroptosis resistance and cell proliferation, IL15 was added to the culture medium at a concentration of 2 pg/mL. Western blot analysis revealed that protein levels of IL15RA, p-STAT3, GPX4 and ACSL3 were elevated in the coculture group as well as in the IL15 treatment group compared with those in the control group ( Figure 4A). BODIPY 581/591 C11 probe was used to detect oxidized lipid, and the results revealed a decrease in lipid peroxidation level after IL15 treatment ( Figure 4B). MDA level was decreased as well ( Figure 4C). Colony formation assay demonstrated higher proliferation ability of pancreatic cancer cells after IL15 treatment ( Figure 4D).

**Figure 5:**
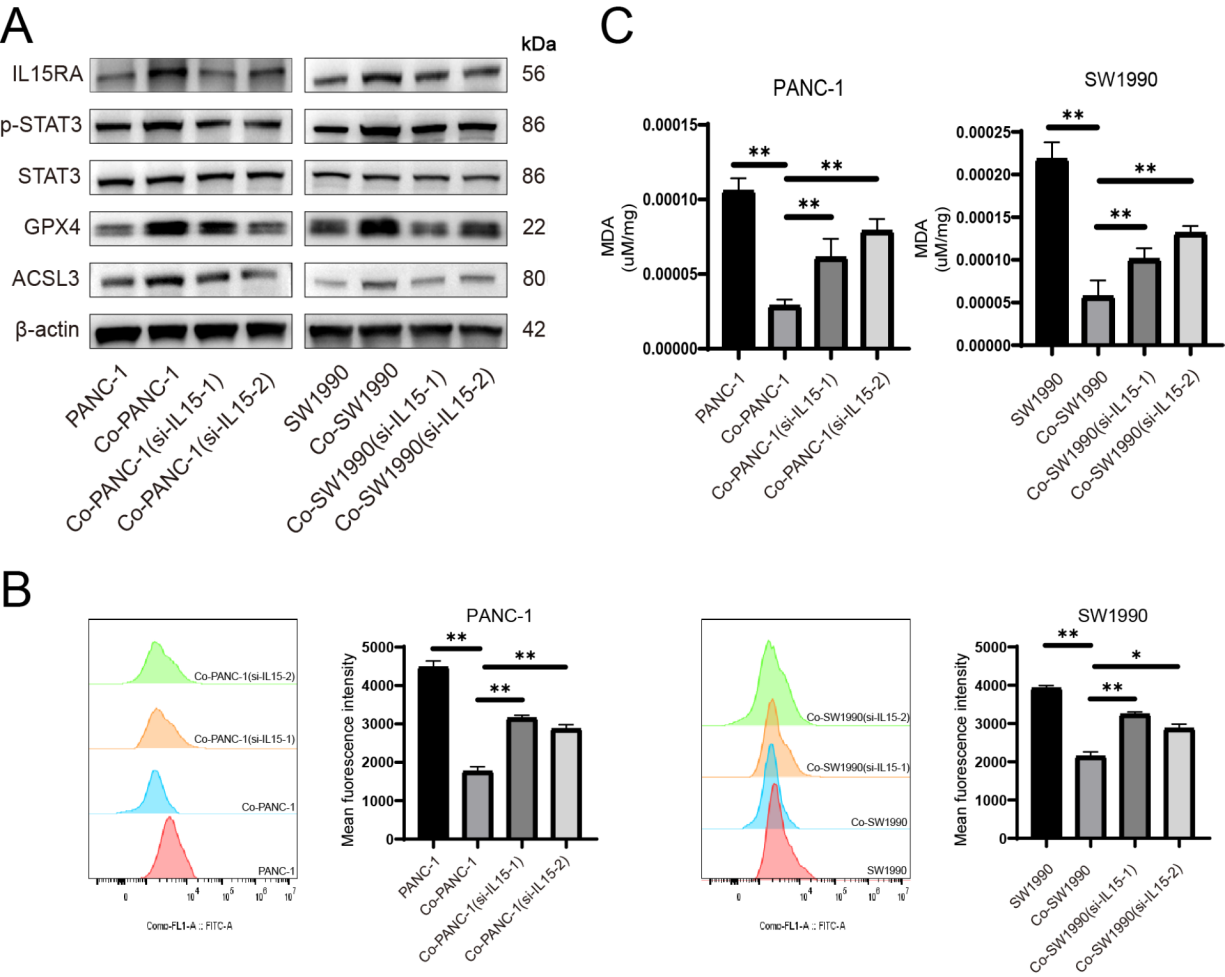
Figure 5 Activated PSCs mediate ferroptosis resistance in pancreatic cancer cells and promote cell proliferation by the secretion of IL15 (A) IL15 in PSCs was silenced before cocultured with pancreatic cancer cells, and protein levels of IL15RA, p-STAT3, STAT3, GPX4 and ACSL3 in pancreatic cancer cells were detected. (B) BODIPY 581/591C11 was used to detect lipid peroxidation level. (C) Concentration of MDA was detected in pancreatic cancer cells. *P < 0.05, **P < 0.01.

Furthermore, we used small interfering RNA to silence *IL15* in activated PSCs and detected whether it affects the protein level of pancreatic cancer cells. *IL15* silencing decreased IL15RA, p-STAT3, GPX4 and ACSL3 expression levels ( Figure 5A). Meanwhile, lipid peroxidation level as well as MDA level were increased after *IL15* silencing ( Figure 5B,C).

### IL15RA mediates the activation of STAT3 and the upregulation of the downstream genes *GPX4* and *ACSL3*

**Figure 6:**
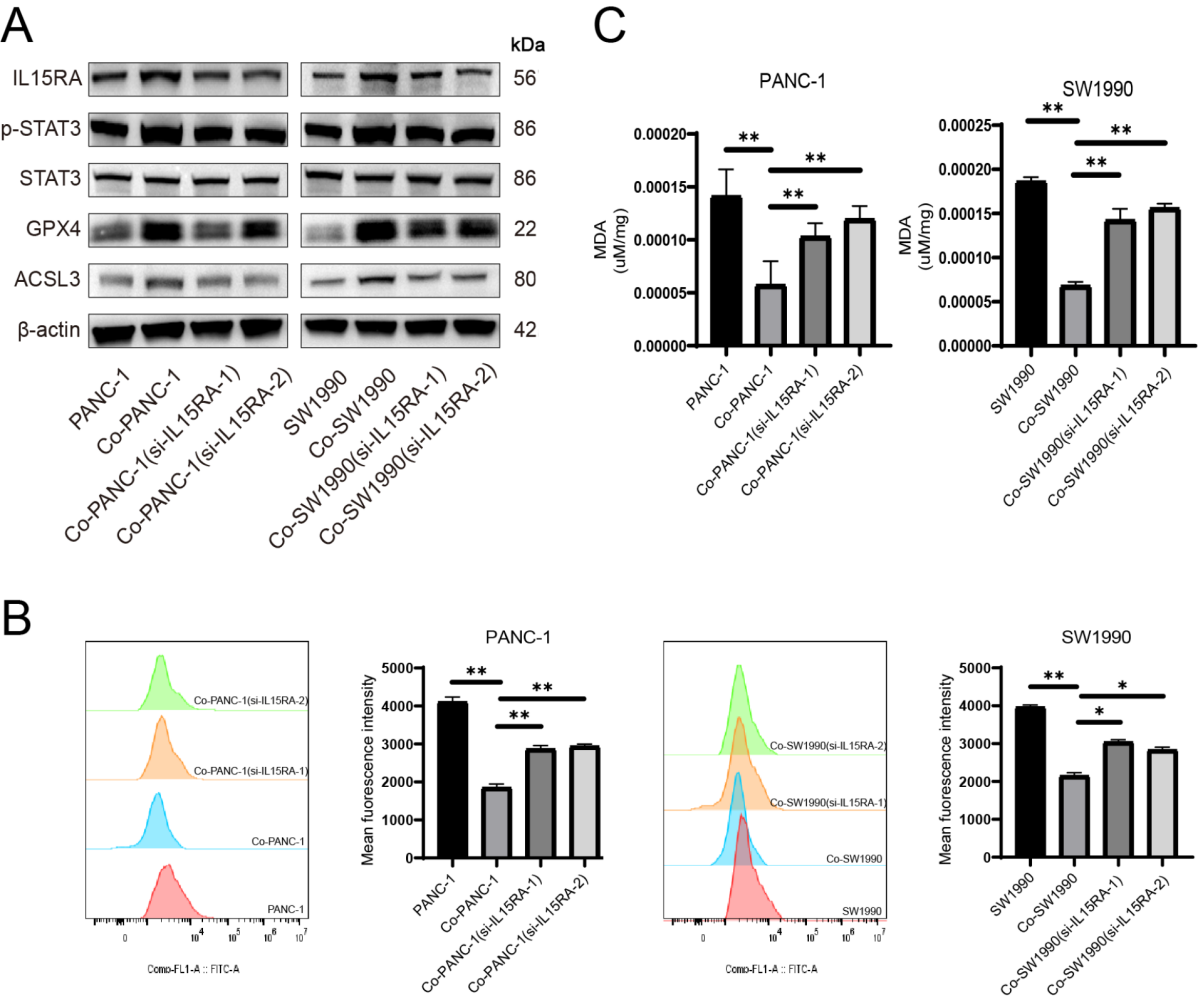
Figure 6 IL15RA mediate ferroptosis resistance in pancreatic cancer cells via activation of STAT3 and upregulation of GPX4 and ACSL3 (A) Protein levels of IL15RA, p-STAT3, STAT3, GPX4 and ACSL3 in pancreatic cancer cells were detected after IL15RA knockdown. (B) BODIPY 581/591C11 was used to detect lipid peroxidation level. (C) Concentration of MDA was detected in pancreatic cancer cells. *P < 0.05, **P < 0.01.

To further analyze whether STAT3 can be activated by IL15RA, we used small interfering RNA to silence *IL15RA* in pancreatic cancer cells. Western blot analysis revealed that p-STAT3, GPX4 and ACSL3 expression levels were decreased after *IL15RA* silencing ( Figure 6A). Similarly, lipid peroxidation level as well as MDA level was increased after *IL15* silencing ( Figure 6B,C). These results indicated that *IL15RA* may be a potential target gene mediating the activation of STAT3 and upregulation of GPX4 and ACSL3 which are of great importance in cellular self-protection against ferroptosis.

### Inhibition of STAT3 decreases GPX4 and ACSL3 expression levels and increases cellular ROS level

**Figure 7:**
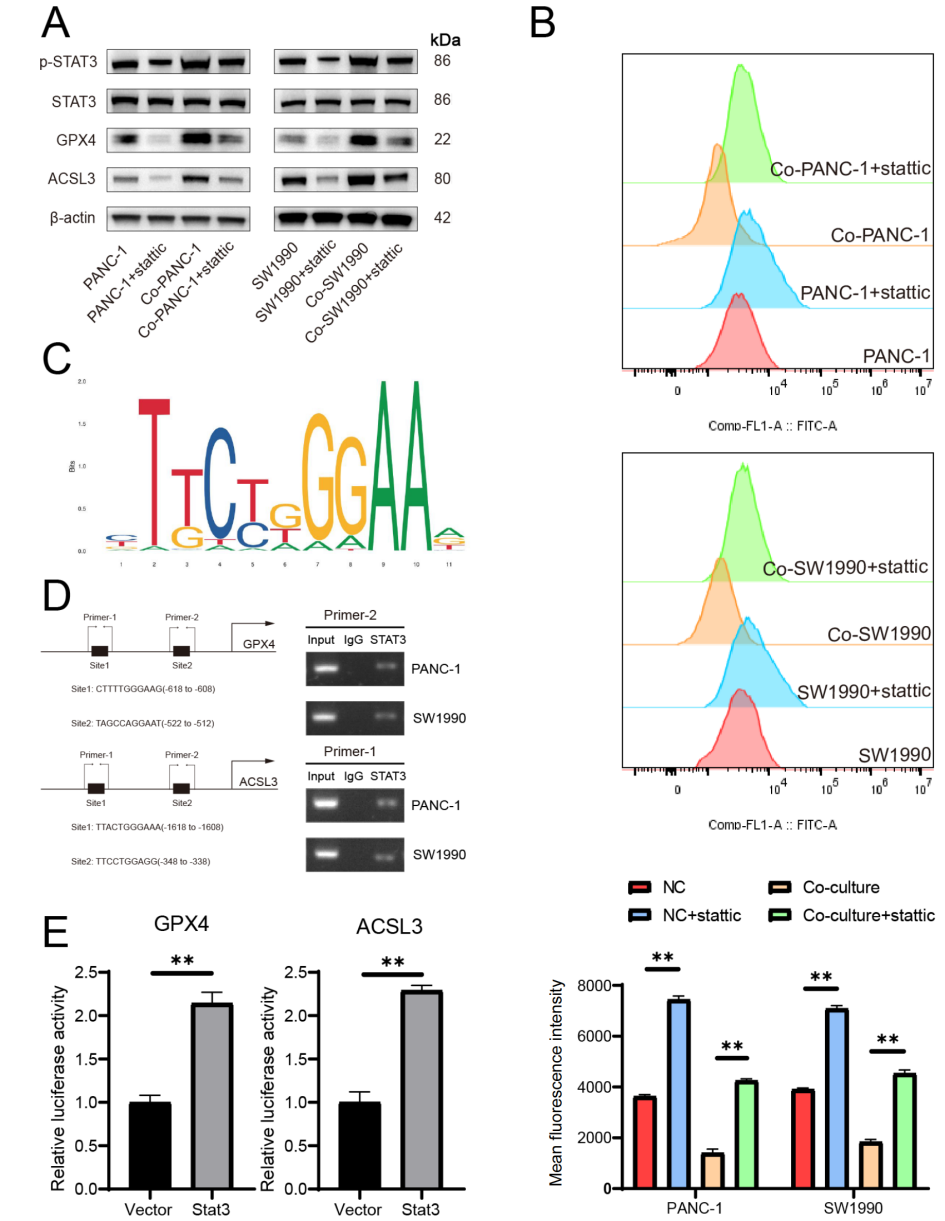
Figure 7 STAT3 promotes GPX4 and ACSL3 expression levels as a transcription factor and mediates ferroptosis resistance (A) Protein levels of p-STAT3, STAT3, GPX4 and ACSL3 in pancreatic cancer cells were detected after Stattic treatment. (B) BODIPY 581/591C11 was used to detect lipid peroxidation level. (C) JASPAR website (http://jaspar.genereg.net/) was utilized to predict the binding sites of STAT3 in the GPX4 and ACSL3 promoter. (D) ChIP assay was conducted with STAT3 antibody. Schematic diagram (left) demonstrating the potential binding site of STAT3 on GPX4 and ACSL3 promoter. Agarose gel electrophoresis (right) was used to confirm the binding site. (E) Dual-luciferase reporter system was used to detect the effect of STAT3 on GPX4 and ACSL3 promoter activity. **P < 0.01.

The small-molecule inhibitor Stattic can reverse STAT3-Y705 phosphorylation. Here, Stattic was added to the culture medium at a concentration of 5 μM to inactivate STAT3. Western blot analysis revealed that, after Stattic treatment, p-STAT3, GPX4 and ACSL3 expression levels were decreased ( Figure 7A). Moreover, a dramatic increase in lipid peroxidation level was observed ( Figure 7B). The JASPAR website ( http://jaspar.genereg.net/) was used to predict the binding sites of STAT3 in the *GPX4* and *ACSL3* promoters ( Figure 7C). We found that STAT3 has several binding sites in the *GPX4* and *ACSL3* promoter regions, and we then conducted a ChIP assay using an anti-STAT3 antibody to verify whether STAT3 binds to the *GPX4* and *ACSL3* promoters. Our results revealed that STAT3 binds to the *GPX4* promoter at the site corresponding to primer 2 and the *ACSL3* promoter at the site corresponding to primer 1 ( Figure 7D). Finally, we designed luciferase reporter gene plasmids containing the *GPX4* and *ACSL3* promoter region sites and transfected them into 293T cells. By comparing the fluorescence values, we found that STAT3 overexpression increased *GPX4* and *ACSL3* promoter reporter activity ( Figure 7E). Collectively, these data suggested that Stattic altered *GPX4* and *ACSL3* transcription by inhibiting STAT3-Y705 phosphorylation and deactivating STAT3.

### Activated PSCs promote tumor growth and *IL15RA* knockout in SW1990 cells inhibits tumor growth

**Figure 8:**
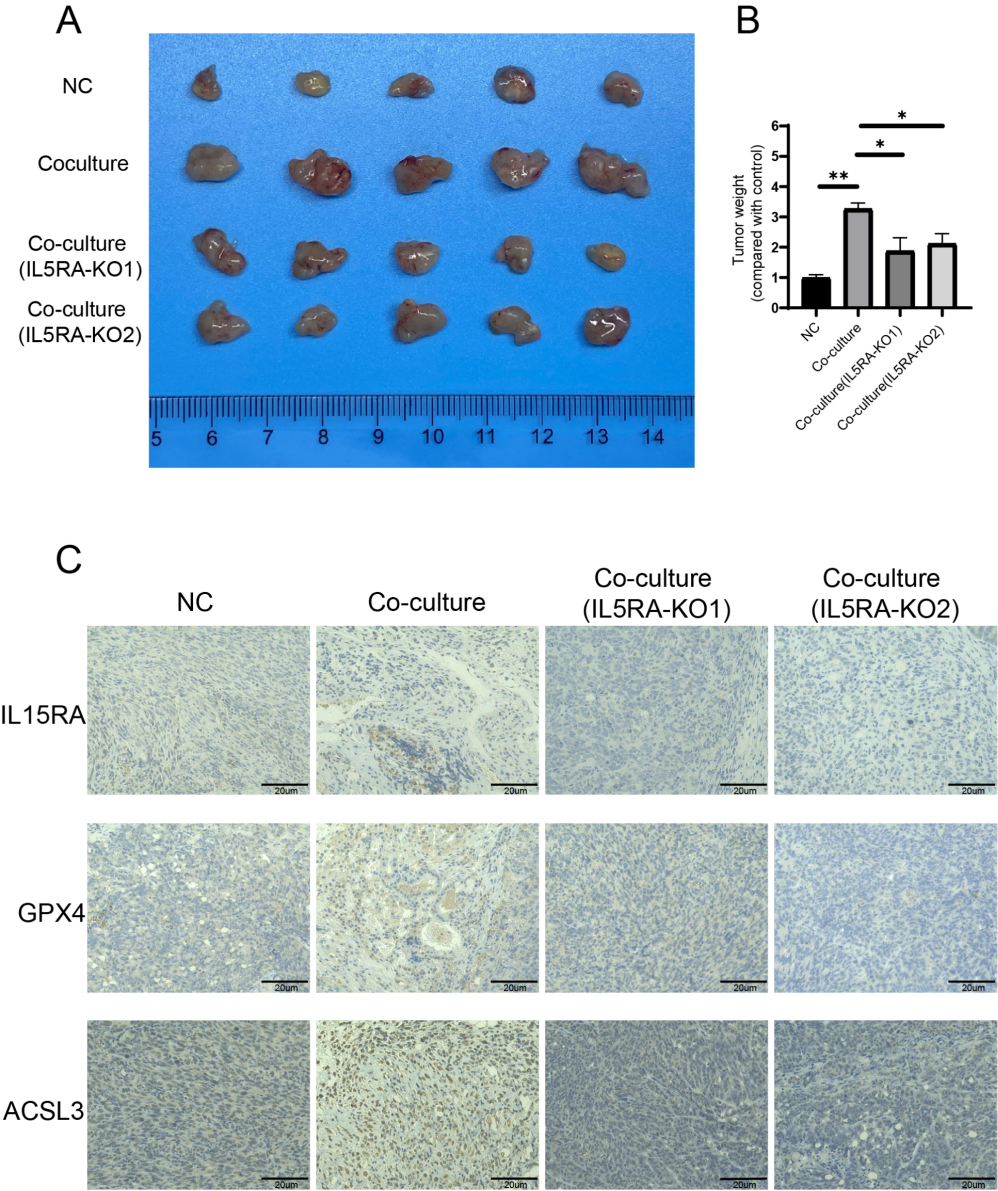
Figure 8 Activated PSCs promote tumor growth and IL15RA increases the protein levels of GPX4 and ACSL3 (A) The average tumor volume in each group was measured and the tumors were photographed. (B) The average tumor weight in each group. (C) Immunohistochemical (IHC) staining of IL15RA, GPX4 and ACSL3 in each group. Scale bar: 20 μm. *P < 0.05, **P < 0.01.

To confirm whether activated PSCs can promote tumor growth *in vivo*, orthotopic combining injection of PSCs and pancreatic cancer cells were performed. The results showed that upon combining injection, tumor size and tumor weight were both increased. Meanwhile, *IL15RA* knockout in SW1990 cells attenuated the combining injection effect ( Figure 8A,B). Next, we examined whether activated PSCs modulate IL15RA expression in SW1990 cells. Our results demonstrated that activated PSCs increased the protein level of IL15RA, and the protein levels of GPX4 and ACSL3 were also increased. However, knockout of *IL15RA* in SW1990 cells impaired the protein levels of GPX4 and ACSL3 ( Figure 8C).

## Discussion

Pancreatic cancer has a poor prognosis because of its lack of effective diagnostic and treatment methods [14]. The five-year survival rate of patients with pancreatic cancer is very low regardless of the presence of resectable pancreatic cancer, locally advanced pancreatic cancer or pancreatic cancer with distant metastasis [15]. In addition, it is estimated that by 2030, pancreatic cancer will become the second leading cause of cancer-related deaths [16].

The complexity of the tumor microenvironment (TME) is one of the main reasons for the intractability of PDAC. The TME component of pancreatic cancer is complex and dynamic, similar to the TME of general tumors. However, pancreatic cancer has its own unique features. PDAC has a high degree of connective tissue proliferation and is rich in PSCs, which can secrete large amounts of extracellular matrix and cytokines to regulate the biological behavior of pancreatic cancer [17].

Interactions between pancreatic cancer and PSCs have been widely reported [ 18‒ 20], as have interactions between ferroptosis and pancreatic cancer [ 21‒ 23]. However, few studies have reported whether PSCs can affect ferroptosis in pancreatic cancer. This study revealed that upon coculture, PSCs can increase the proliferative ability and ferroptosis resistance of pancreatic cancer cells. To further analyze the mechanisms underlying this phenomenon, RNA-Seq was performed to search for potential downstream genes that might mediate changes in proliferation ability and ferroptosis resistance. Among the multiple genes, *IL15RA* has attracted our attention. *IL15RA* encodes a cytokine receptor that binds to IL15 with high affinity and specificity. IL15RA on the surface of pancreatic cancer cells is upregulated after coculture. We suspect that PSCs secrete IL15 and then activate the receptor on the surface of the pancreatic cancer cell membrane. Previous studies have shown that IL15 may be used for immunotherapy in pancreatic cancer. Owing to its ability to activate IL15RA on the membrane surface of effector cells such as T cells and natural killer (NK) cells [ 24, 25], it is widely recognized as an anticancer cytokine. Moreover, IL15RA is expressed in various cells, including malignant tumor cells [ 26, 27]. After binding with its ligand IL15, IL15RA expressed by malignant tumor cells presents it to NK and T cells, activating effector cells and further attacking malignant tumor cells [ 28‒ 31]. Therefore, IL15 is considered a cytokine for treating cancer. This seems to contradict our results. However, the coculture system in this study contained only pancreatic stellate cells and pancreatic cancer cells but not immune cells. Therefore, we suspect that IL15 can be used as a cancer-promoting factor to promote the growth of pancreatic cancer cells. Through a literature review, we found that in the absence of immune cells, IL-15RA on the surface of gastric cancer cells induces a malignant phenotype, including increased cell growth, migration and invasion and decreased apoptosis [32]. Therefore, we added IL15 to pancreatic cancer cell culture medium, and the results confirmed that IL15 can promote the proliferation and ferroptosis resistance of pancreatic cancer cells.

In this study, the coculture system included only pancreatic stellate cells and pancreatic cancer cells both *in vitro* and *in vivo*. The establishment of this model has certain limitations. There are a variety of cell types in the tumor microenvironment of pancreatic cancer, including immune cells. On the one hand, the IL-15 secreted by pancreatic stellate cells protects pancreatic cancer cells by improving ferroptosis resistance; on the other hand, the IL-15 secreted by pancreatic stellate cells can also activate T cells and NK cells to play an antitumor immune role. Therefore, whether IL-15 secreted by pancreatic stellate cells *in vivo* can protect pancreatic cancer cells remains to be verified by further animal experiments.

In conclusion, PSCs protect pancreatic cancer cells from ferroptosis by secretion of IL15, which further activates the IL15R-STAT3-GPX4/ACSL3 axis in a paracrine way.

## Supporting information

24207supplementary_Figures

## Supplementary Data

Supplementary data is available at *Acta Biochimica et Biophysica Sinica* online.
