# Supplementary material for: IL15RA-STAT3-GPX4/ACSL3 signaling leads to ferroptosis resistance in pancreatic cancer: IL15RA-STAT3-GPX4/ACSL3 leads to ferroptosis resistance in PC
Source: Acta Biochim Biophys Sin (Shanghai). 2024 Oct 12;57(3):389–402. doi: 10.3724/abbs.2024153 (PMC11986442; doi:10.3724/abbs.2024153)
Supplement: 24207supplementary_Figures [file 24207supplementary_Figures.docx]

**
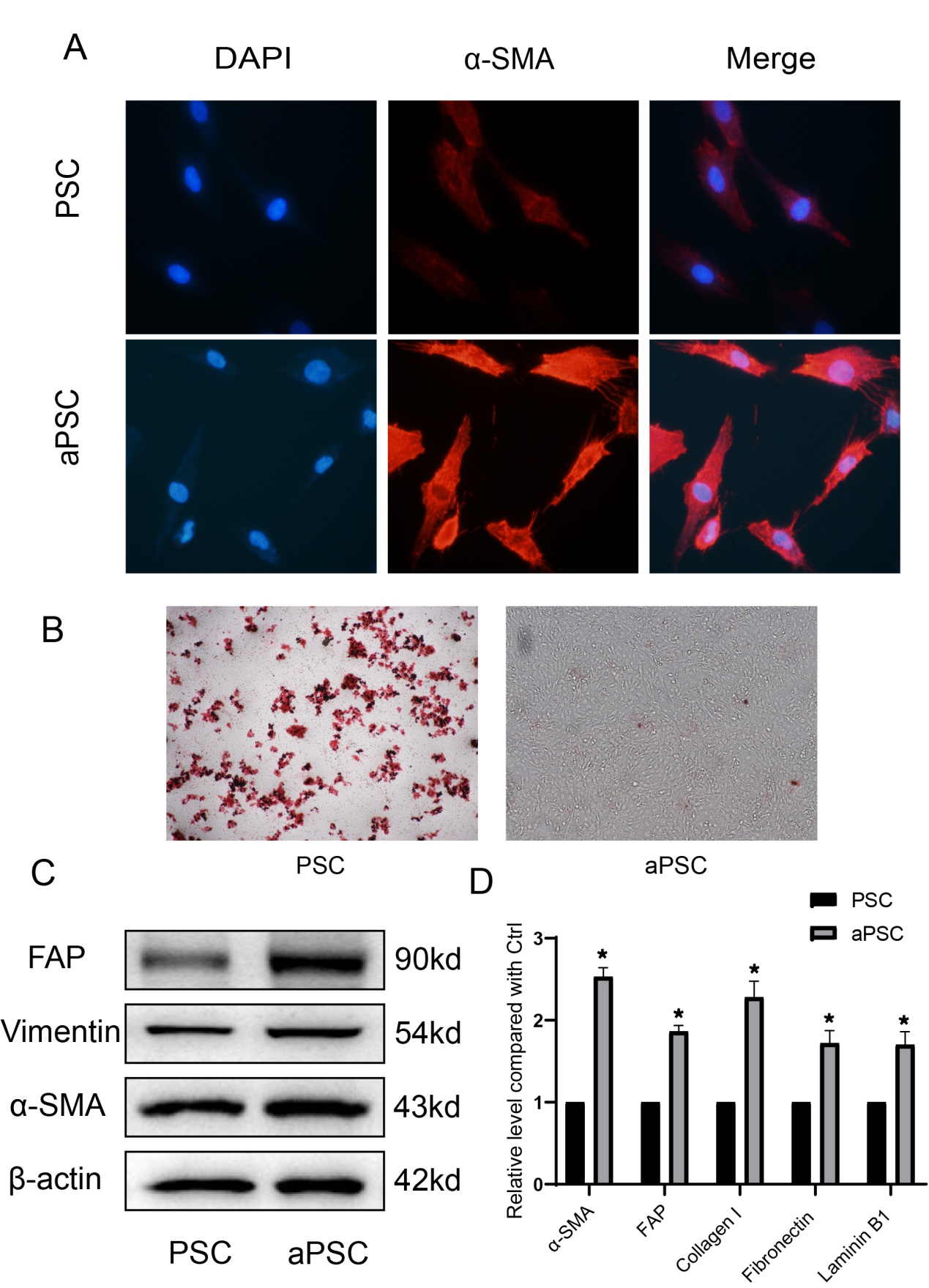
**

**Supplementary Figure S1. PSCs could be activated by pancreatic cancer cells upon coculture** (A) Cellular immunofluorescence of α-SMA in PSCs. (B) Oil red staining of PSCs with or without coculture of pancreatic cancer cells. (C) Protein levels of FAP, Vimentin and α-SMA were detected by western blot analysis. (D) qRT‒PCR detected the mRNA levels of *α-SMA*, *FAP*, *Collagen I*, *Fibronectin* and *Laminin B1*.

**
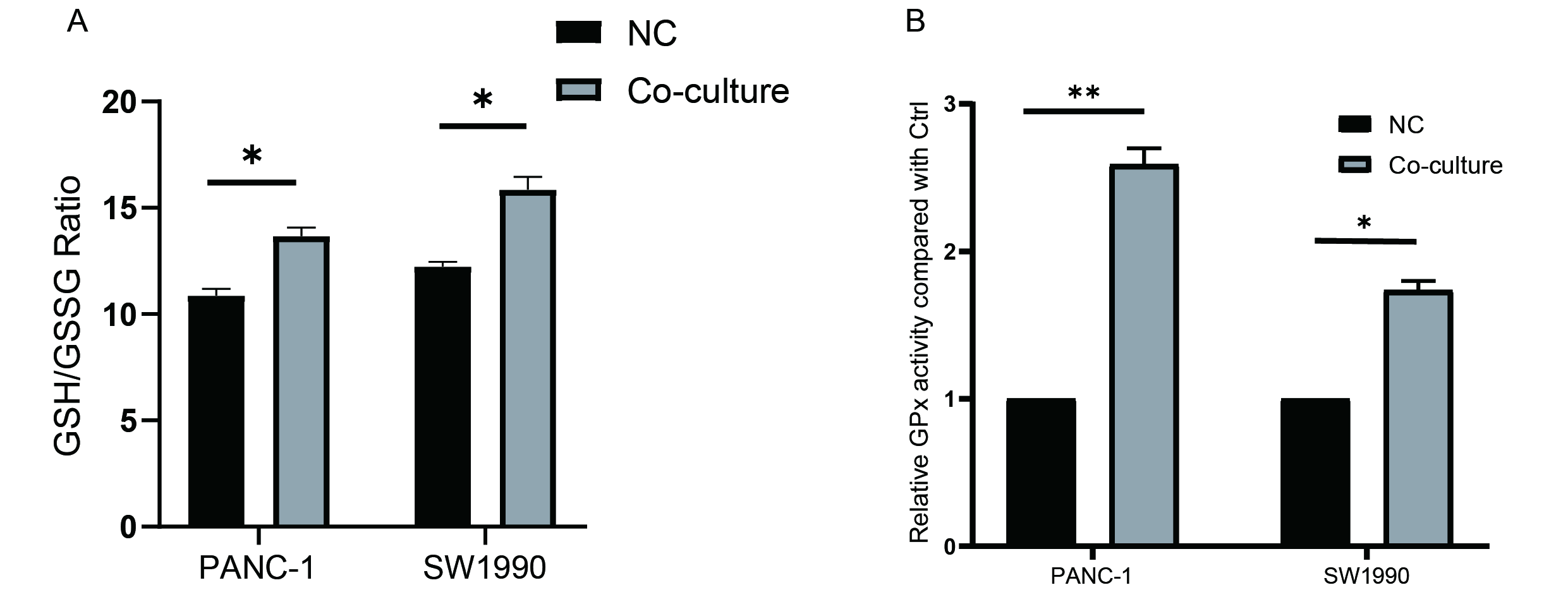
**

**Supplementary Figure S2. Activated PSCs could increase the GSH/GSSG ratio and GPx activity of pancreatic cancer cells** (A) GSH/GSSG ratio of pancreatic cancer cells with or without coculture of PSCs. (B) GPx activity of pancreatic cancer cells with or without coculture of PSCs.


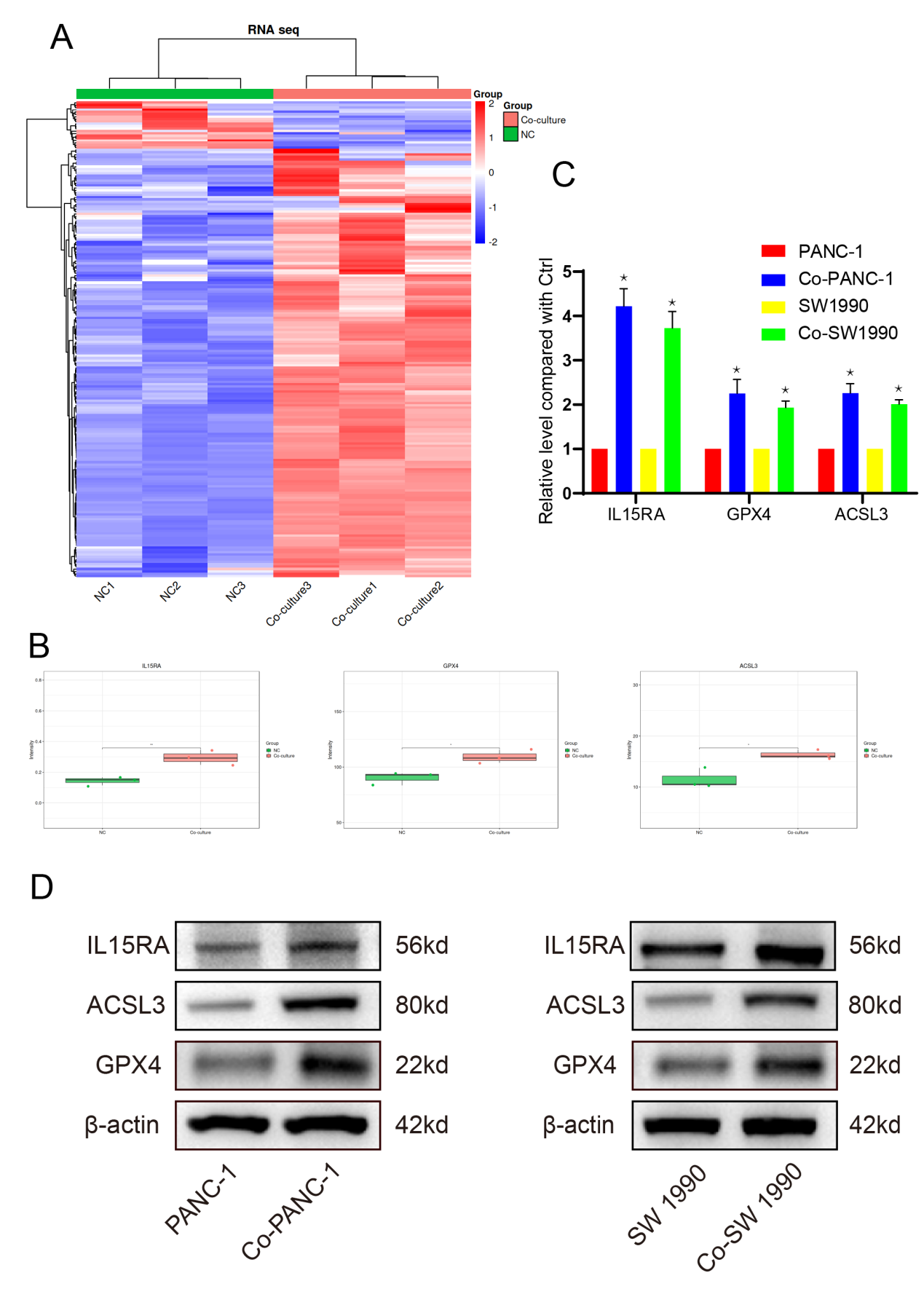


**Supplementary Figure S3. The expression levels of IL15RA, ACSL3 and GPX4 in pancreatic cancer cells were upregulated upon cocultured with activated PSCs** (A) Heatmap demonstrated that the number of upregulated genes was much more than the number of downregulated genes in pancreatic cancer cells upon coculture. (B) Boxplots demonstrated the expression levels of IL15RA, ACSL3 and GPX4. (C,D) qR-PCR and western blot analysis detected the mRNA and protein levels of IL15RA, ACSL3 and GPX4.

**
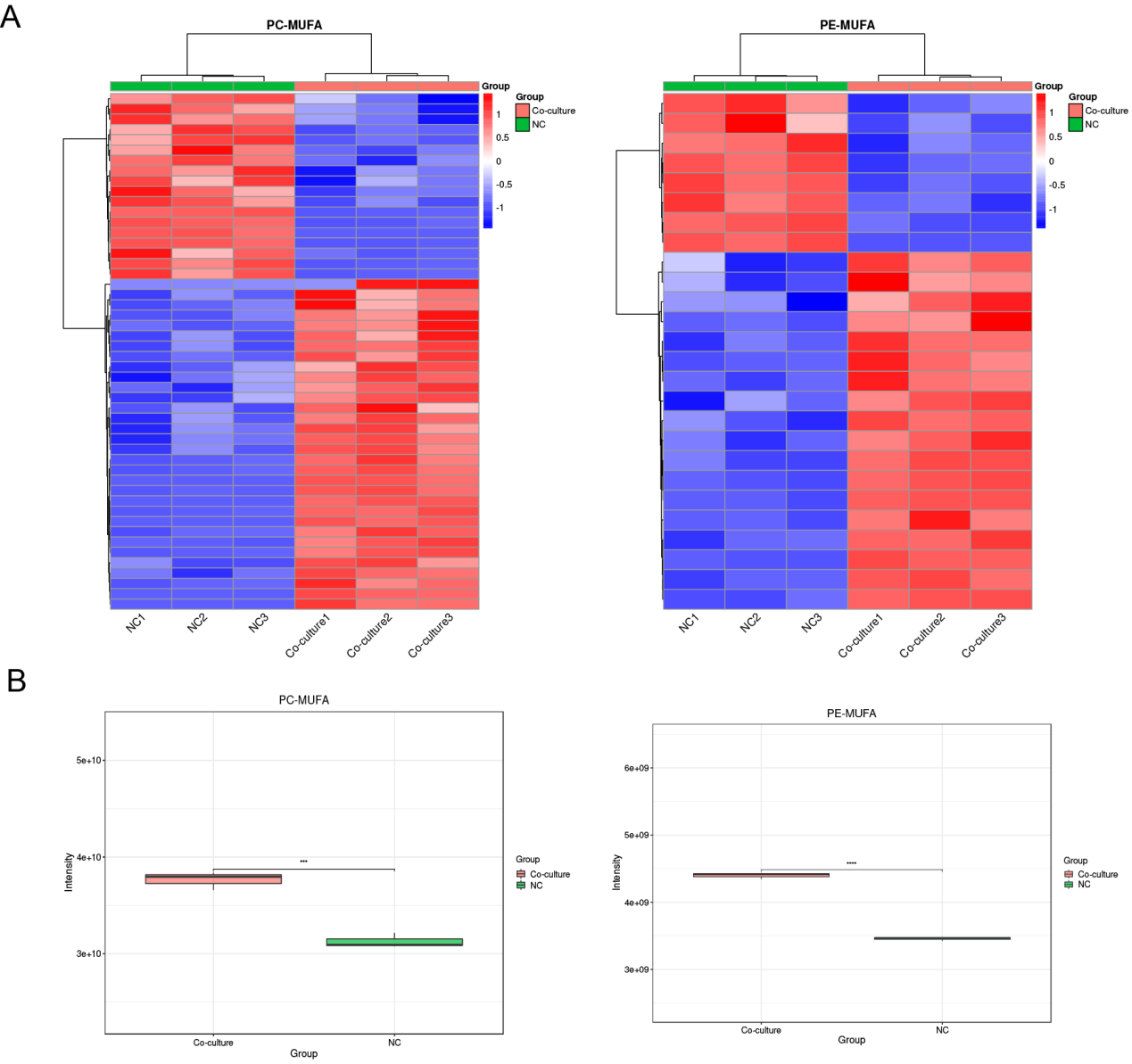
**

**Supplementary Figure S4. Activated PSCs increased the content of PC-MUFAs and PE-MUFAs in pancreatic cancer cells and promoted gemcitabine resistance**  (A,B) The contents of PC-MUFAs and PE-MUFAs were tested.


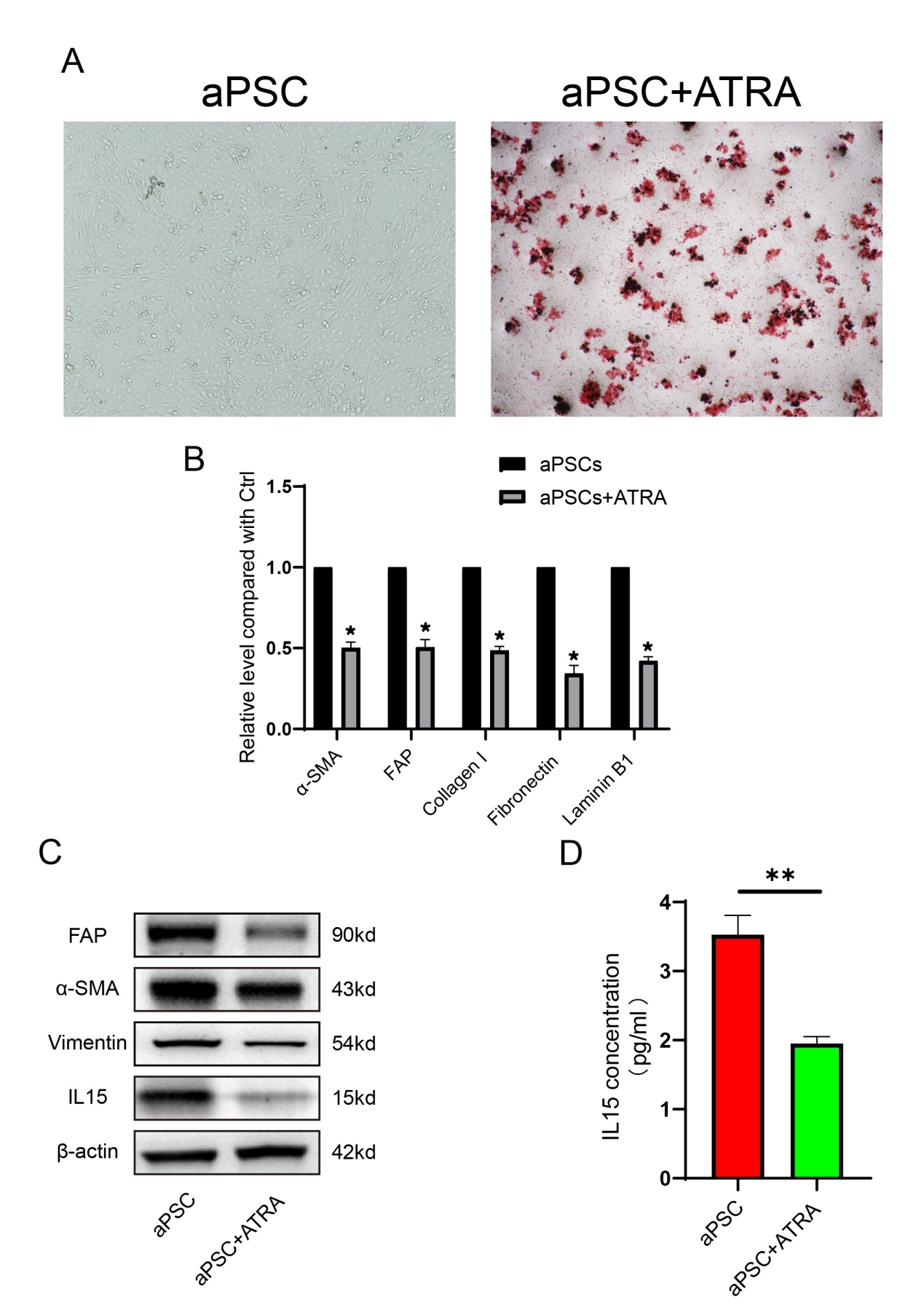


**Supplementary Figure S5. ATRA could reverse the activation state of PSCs and reduce the secretion of IL15** (A) Oil red staining was performed to observe lipid droplets after ATRA treatment. (B) qRT‒PCR detected the mRNA levels of *α-SMA*, *FAP*, *Collagen I*, *Fibronectin* and *Laminin B1*. (C) Protein levels of FAP, Vimentin, α-SMA and IL15 were detected by western blot analysis. (D) IL-15 levels in the culture supernatants were measured using the Human IL-15 ELISA Kit.
